# Metabolic Effects of Bee Larva-Derived Protein in Mice: Assessment of an Alternative Protein Source

**DOI:** 10.3390/foods10112642

**Published:** 2021-11-01

**Authors:** Yoko Yokoyama, Kawori Shinohara, Naho Kitamura, Anna Nakamura, Ai Onoue, Kazuki Tanaka, Akiyoshi Hirayama, Wanping Aw, Shigeru Nakamura, Yoko Ogawa, Shinji Fukuda, Kazuo Tsubota, Mitsuhiro Watanabe

**Affiliations:** 1 Graduate School of Media and Governance, Keio University, Fujisawa 252-0882, Kanagawa, Japan; yyokoyama-kyt@umin.ac.jp (Y.Y.); insect.kawo@gmail.com (K.S.); nahoshi@sfc.keio.ac.jp (N.K.); anna87@sfc.keio.ac.jp (A.N.); tkazu@sfc.keio.ac.jp (K.T.); 2 Health Science Laboratory, Keio Research Institute at SFC, Fujisawa 252-0882, Kanagawa, Japan; tsubota@tsubota-lab.com; 3 Department of Environment and Information Studies, Keio University, Fujisawa 252-0882, Kanagawa, Japan; t18185ao@sfc.keio.ac.jp; 4 Institute for Advanced Biosciences, Keio University, Tsuruoka 997-0052, Yamagata, Japan; hirayama@ttck.keio.ac.jp (A.H.); wanping@sfc.keio.ac.jp (W.A.); sfukuda@sfc.keio.ac.jp (S.F.); 5 Gut Environmental Design Group, Kanagawa Institute of Industrial Science and Technology, Kawasaki 210-0821, Kanagawa, Japan; 6 Department of Ophthalmology, Keio University School of Medicine, Shinjuku, Tokyo 160-8582, Japan; vdtwork@gmail.com (S.N.); yoko@z7.keio.jp (Y.O.); 7 Transborder Medical Research Center, University of Tsukuba, Tsukuba 305-8575, Ibaraki, Japan; 8 Tsubota Laboratory, Inc., Shinjuku, Tokyo 160-0016, Japan; 9 Department of Internal Medicine, Keio University School of Medicine, Shinjuku, Tokyo 160-8582, Japan

**Keywords:** edible insects, protein, nutrition balance, metabolite, bee larva

## Abstract

Food crises caused by growing global population or environmental changes are predicted in the near future; therefore, sustainable solutions are needed. Edible insects, which are rich in protein and can save feed and environmental resources, have the potential to be a sustainable alternative protein source. However, there is limited evidence on the impact on health. In this study, we investigated the biological effects of ingesting bee larva by examining their effects on amino acid, lipid, and glucose metabolism in animal models. In our animal experiments, the replacement of casein as a protein source, with edible insects, did not seem to cause any deficiency in murine amino acid levels in the plasma and liver. Metabolomic analysis of plasma metabolites showed decreased 3-methylhistidine and increased nicotinamide in the bee larva-derived protein-fed mice. Decreased levels of plasma 3-metylhistidine, an indicator of muscle degradation, implies that replacement to bee-larva protein from casein did not cause muscle degradation in vivo. We further investigated effects of increased plasma nicotinamide on peripheral tissue and found an increase in expression levels of genes involved in glucose uptake in muscle and thermogenesis in adipose tissue. These data imply that bee larva is a potential sustainable, safe and healthy alternative protein source.

## 1. Introduction

Protein is an essential nutrient for living organisms and is involved in a wide range of metabolic activities [1]. The global population is expected to reach 10 billion by 2050, and protein production to meet this demand sufficiently has become a global issue [2]. Food production is one of the largest causes leading to detrimental global environmental changes [2]. For example, food production uses 40% of global land [3], is responsible for 30% of global greenhouse-gas emissions [4] and uses 70% of fresh water [5,6]. Therefore, it has become important to find sustainable and healthy alternative protein sources [7,8].

According to the Food and Agriculture Organization of the United Nations (FAO), edible insects are attracting attention as a solution to the food crisis and environmental destruction because of their high feed conversion rate, which allows the same amount of protein to be obtained from 25% of the feed as beef. In addition, less methane, ammonia, global warming greenhouse gases and manure are generated, which can reduce environmental pollution [8,9,10].

Japan has the lowest rates of obesity and the highest rates of longevity in the world [11,12]. Japan is one of the few countries where edible insects are consumed [13], and over 50 species of insects have been recorded as edible [14]. Previously, edible insects have been used as compensation for food shortages [15,16,17].

One of them, bee larvae, has been traditionally consumed in Japan [18] and is a rich source of protein, lipids, sugars, vitamins, amino acids and numerous minerals [19]. According to the researchers, honeybee brood, larvae and pupae of drones may be a more sustainable alternative protein source than other edible insects [20]. The authors note that in some parts of the world, drone brood removal has been part of regular hive maintenance by beekeepers as a management strategy for populations of varroa mite (*Varroa destructor*), widely considered to be the most harmful parasite to honey bees [21]. In this method, bee larvae are produced as a by-product, providing a rich source of farmed insects with unknown potential [20]. The cultivation of bee larvae has many advantages, including the relatively small amount of cultivated land required to set up a hive and the low economic investment required. In addition, research on the ecology and reproduction of honeybees has a longer history than other candidates for insect farming [20]. Therefore, whereas honeybee larvae may not be a replacement for other protein value chains, honeybee larvae could still exist as a feasible addition to a more sustainable food industry. Further research is required to confirm the sustainability of bee larvae as a potential complementary source of protein in the future.

Previous studies have shown that differences in dietary protein sources alone can alter the metabolic regulatory mechanisms of the organism and contribute to changes in the risk of developing metabolic diseases such as obesity and diabetes [22,23,24], muscle mass maintenance [25] and mortality [26]. Therefore, in the dissemination of a potentially sustainable protein, the effect on the metabolic regulatory mechanism of the body when traditional protein is replaced should be carefully considered. In the present study, we examined the effects on metabolic control mechanisms when all dietary protein sources were replaced by bee larva protein using a mouse model to investigate its impact on metabolism [23].

However, although research on the composition of these edible insects is progressing [27], there is a lack of evidence on their effects in vivo [28,29]. The safety and effects of edible insects in vivo needs to be clarified in order to establish potential alternative proteins to solve the world’s protein production.

Therefore, the purpose of this study is to clarify the safety of bee larva protein as an alternative source of protein, and to understand its effectiveness in vivo using comprehensive metabolite analysis.

## 2. Materials and Methods

### 2.1. Animal Experiments

Animal experiments were approved by the Institutional Animal Care and Use Committee of Keio University (Ethical code 14071). All sacrifices were performed under isoflurane anesthesia. Five-week-old C57BL/6J male mice were purchased from CLEA Japan, Inc. (Shizuoka, Japan). The mice were kept in a facility with proper control of the temperature, humidity, and lighting (12 h light/dark cycle). After one week of acclimatization, the animals were divided into three groups: (1) 15% kcal casein protein diet (15% casein), (2) 30% kcal casein protein diet (30% casein) and (3) 30% kcal protein bee larva diet (30% bee). The 15% kcal casein protein group was added to compare the results with those of the average intake, according to the National Health and Nutrition Examination Survey (NHANES) [30,31]. Body weight and food intake were measured weekly. After 22 weeks of treatment, the animals were fasted for 4 h before dissection. Plasma, liver, white adipose tissue, brown adipose tissue, heart and muscles were collected and stored at −80 °C until further analysis.

### 2.2. Ingredients of the Diets

Freeze-dried bee larvae powder (approximately 21 days old) was obtained from the Yamada Bee Company, Inc. (Okayama, Japan). The composition of bee larva powder is 48.5 g protein, 19.4 g carbohydrate, and 20.8 g fat per 100 g (Yamada Bee Company, Inc, Okayama, Japan). The ingredients of the diets are shown in Supplemental Table S1. Cornstarch was adjusted for the carbohydrate content of the protein sources, ensuring that the % energy from carbohydrates remained the same for the diets, according to the previous study [23].

### 2.3. Oral Glucose Tolerance Test

An oral glucose tolerance test was performed at 12 weeks of treatment; after an overnight fast, glucose was administered orally at a dose of 1.5 g/kg body weight. Blood samples were collected from the tail vein at baseline and at 15, 30, 60, 120 and 180 min. Blood glucose concentrations were measured using the Life Check (GUNZE Limited, Osaka, Japan). Plasma insulin levels were measured using enzyme-linked immunosorbent assay (ELISA, Morinaga Institute of Biological Science, Inc., Kanagawa, Japan) according to the manufacturer’s instructions.

### 2.4. Wire Hang Test

The animal was placed on the cage top, which was then inverted and hung above the home cage, and the time until the animal falls was recorded.

### 2.5. Analysis of mRNA Gene Expression

Total RNA was extracted using the RNeasy Mini Kit (Qiagen, Hilden, Germany). The Prime Script^®^ Master Mix (TaKaRa, Shiga, Japan) was used to synthesize template cDNA for real-time PCR from total RNA. Gene fragments of interest were amplified by real-time PCR using SYBR^®^ Premix EX Taq II (TaKaRa, Shiga, Japan) and THUNDERBIRD SYBR qPCR Mix (Toyobo, Osaka, Japan), and each gene expression level was calculated using β-actin, as an internal control to normalize gene expression. List of primers used in this study are shown in Supplemental Table S2.

### 2.6. Metabolite Extraction and Capillary Electrophoresis Time-of-Flight Mass Spectrometry (CE-TOF/MS)-Based Metabolome Analysis

The levels of extracted metabolites were measured in both positive and negative modes by using CE-TOF/MS as previously described [32]. All CE-TOF/MS experiments were performed using a capillary electrophoresis system (Agilent Technologies, Santa Clara, CA, USA). Annotation tables were produced from the measurement of standard compounds and were aligned with the datasets according to similar *m*/*z* values and normalized migration times. Peak areas were normalized against those of the internal standards, methionine sulfone and D-camphor-10-sulfonic acid for cationic and anionic metabolites, respectively. Concentrations of each metabolite were calculated based on their relative peak areas and concentrations of standard compounds. Statistical analysis was performed using Metaboanalyst [33].

### 2.7. Histological Analysis and Transmission Electron Microscopy (TEM)

To perform hematoxylin and eosin (H&E) staining, the collected brown adipose tissue was immediately fixed in 10% neutral buffered formalin. Tissue sections were embedded in paraffin. The staining procedures were performed using standard methods and scanned at ×40 using a Nano Zoomer XR (Hamamatsu Photonics K.K., Shizuoka, Japan). For electron microscopic analysis, the collected brown adipose tissue was immediately fixed using 2.5% glutaraldehyde in 0.1 M phosphate buffer (pH 7.4). To prepare tissue sections, the samples were dehydrated in ethanol, embedded in epoxy resin and then sliced using a microtome equipped with a diamond knife. Uranyl acetate and lead citrate were prepared and used to stain the tissue sections collected on a mesh grid. Brown adipose tissue morphology and mitochondria were observed using an electron microscope (1230 EXII; JEOL, Tokyo, Japan). Images of the tissue sections were captured using a Gatan Bioscan camera model 792.

### 2.8. Statistical Analysis

Comparisons between more than two groups were performed using analysis of variance (ANOVA) and post hoc test. GraphPad Prism 8 (GraphPad Software) was used for all the other statistical analyses. Statistical significance was set at * *p* < 0.05, ** *p* < 0.05, *** *p* ≤ 0.01, and **** *p* ≤ 0.001 unless stated otherwise.

## 3. Results

### 3.1. Bee Larva Protein-Fed Mice Did Not Decrease Body Weight

**Figure 1:**
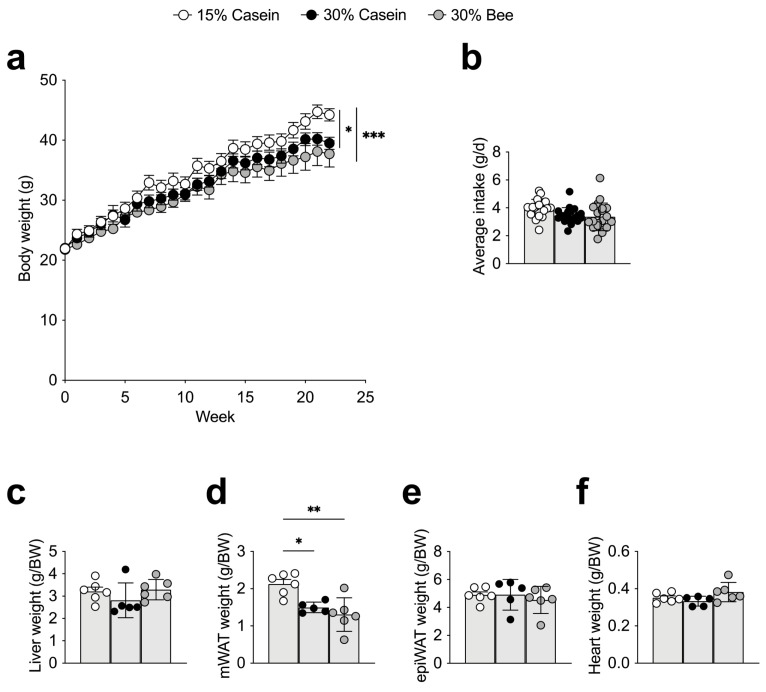
Effects of bee larva protein on body weight, food intake and tissue weights. (**a**–**f**), Male C57BL/6J mice at five weeks of age were fed with a 15% casein diet (15% kcal protein from casein, 30% kcal fat; *n* = 5), a 30% casein diet (30% kcal protein from casein, 30% kcal fat; *n* = 6), or a 30% bee diet (30% kcal protein from bee larva, 30% kcal fat (*n* = 6), for 22 weeks. (**a**) Body weight gain across different groups. (**b**) Average food intake. (**c**) Liver tissue weight per body weight. (**d**) Mesenteric white adipose tissue (mWAT) weight per body weight. (**e**) Epididymal white adipose tissue (epiWAT) weight per body weight. (**f**) Heart weight per body weight. Data are mean ± s.e.m. * *p* ≤ 0.05, ** *p* ≤ 0.01, *** *p* ≤ 0.001. *p* values were calculated using ANOVA and post hoc tests.

Compared with 15% casein-fed mice, 30% casein- (*p* = 0.026) or 30% bee-fed mice (*p* = 0.0006) had significantly reduced body weight, but this did not differ between groups fed with different protein sources (Figure 1a). Dietary intake did not differ between the groups (Figure 1b). Several tissue weights per body weight were compared, and it was found that mesenteric white adipose tissue was reduced in 30% casein- (*p* = 0.018) or 30% bee-fed mice (*p* = 0.002) compared with 15% casein-fed mice, implying that protein quantity was responsible for this difference (Figure 1c–f). Those results imply that bee larva protein did not show toxicity in mice during the 22-week intervention.

### 3.2. Bee Larva Protein Did Not Cause Amino Acids Deficiency in Plasma and Liver

**Figure 2:**
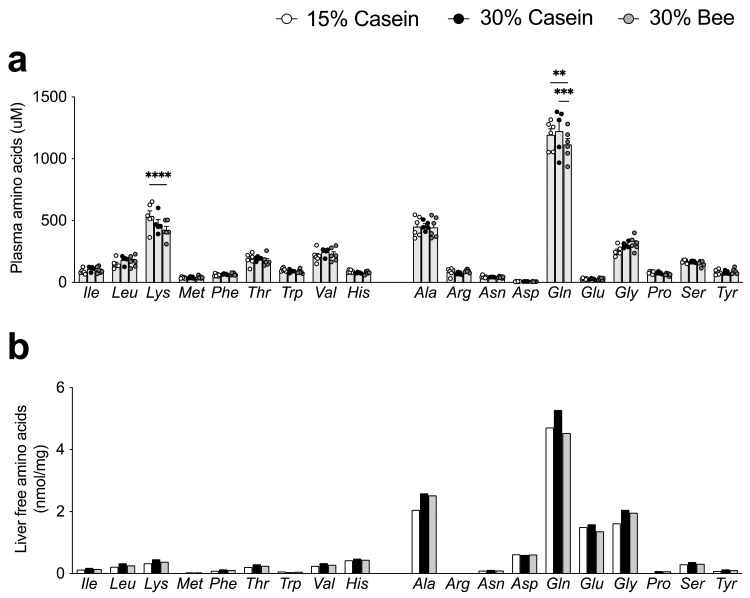
Effects of bee larva protein on amino acids metabolism. (**a**,**b**), Analysis of the mice used in Figure 1. (**a**) Levels of plasma amino acids. (**b**) Levels of liver amino acids. The amino acids were measured in pooled liver samples per treatment group (*n* = 5–6/group). Data are mean ± s.e.m. ** *p* ≤ 0.01, *** *p* ≤ 0.001, **** *p* ≤ 0.0001. *p* values were calculated using ANOVA and post hoc tests.

We examined how bee larvae-derived proteins affected plasma and liver amino acid levels in vivo. The evaluation of plasma essential amino acid levels showed that Lys was significantly lower in mice fed 30% bee than in 15% casein (*p* < 0.0001), but it was not different between 30% bee- and 30% casein-fed mice (Figure 2a). We next examined amino acid levels in the liver, where proteins are synthesized. However, decreased Lys levels in 30% bee were not found in the liver, implying that Lys was not deficient (Figure 2b). Among non-essential amino acids in plasma, Gln was lower in 30% bee-fed mice than in 30% casein-or 15% casein-fed mice, but other amino acid levels were similar among the different diet groups (Figure 2a).

### 3.3. Bee Larva Protein Changed Plasma Metabolites

**Figure 3:**
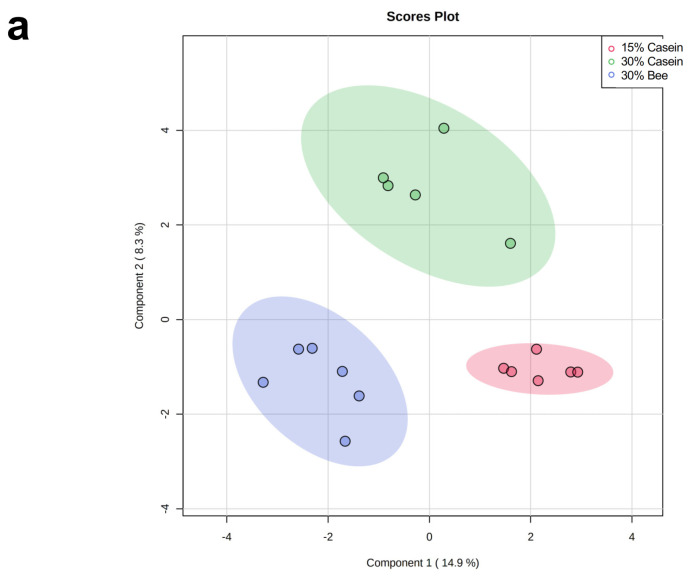

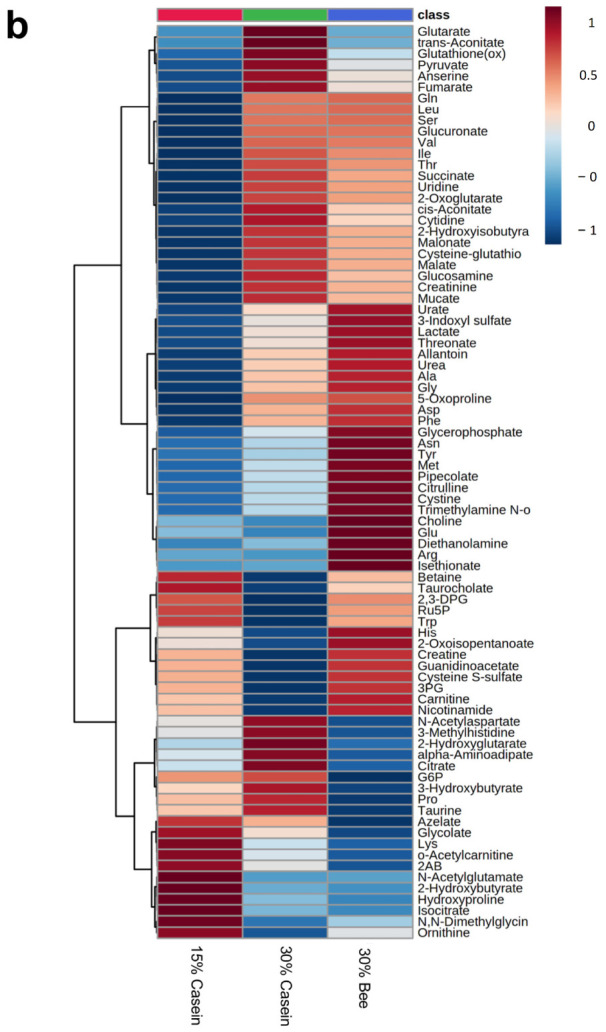

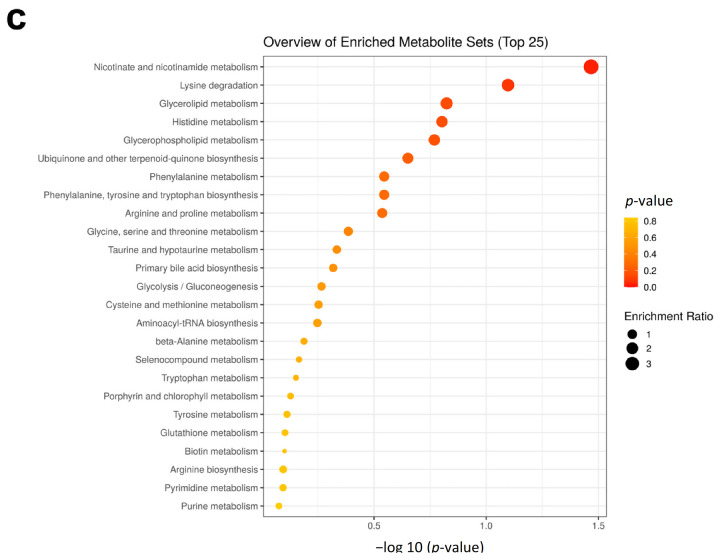
Effects of bee larva protein on plasma metabolite concentrations. (**a**–**c**), Analysis of the mice used in Figure 1. (**a**) The sparse PLS-DA (sPLS-DA) analysis. (**b**) Heatmap to visualize differences in average metabolites among the different diets. (**c**) Enrichment analysis of plasma metabolites compared to 30% Bee larva protein and 30% casein.

To understand the effects of bee larva in vivo, we analyzed the plasma metabolites by CE-TOF/MS. The sparse PLS-DA (sPLS-DA) found that groups were separated depending on different diets (Figure 3a). Heatmap analysis found that metabolites in 30% bee-fed mice were different, compared with 30% casein-fed mice (Figure 3b). The enrichment analysis found that nicotinate and nicotinamide metabolism and some amino acid-related metabolism was enriched in 30% bee-fed mice in comparison with 30% casein-fed mice (Figure 3c).

### 3.4. Plasma 3-Methylhistidine Was Decreased in Bee Larva Protein-Fed Mice

**Figure 4:**
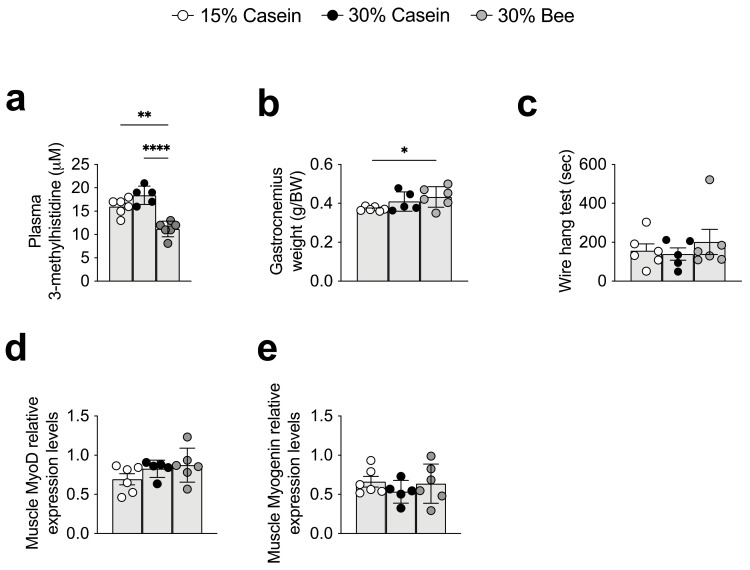
Effects of bee larva protein on muscle degradation and regeneration. (**a**–**e**), Analysis of the mice used in Figure 1. (**a**) Plasma 3-methylhistidine levels. (**b**) Gastrocnemius weight per body weight. (**c**) Wire hang test. The duration (seconds) for which mice remained hanging. (**d**) Relative mRNA expression levels of *MyoD*. (**e**) Relative mRNA expression levels of *Myogenin*. Data are mean ± s.e.m. * *p* ≤ 0.05, ** *p* ≤ 0.01, **** *p* ≤ 0.0001. *p* values were calculated using ANOVA and post hoc tests.

One of the decreased plasma metabolites of 30% bee-fed mice was 3-methylhistidine. 3-methylhistidine, which increases during muscle degradation, was decreased in 30% bee-fed mice compared with 30% casein (*p* < 0.0001) or 15% casein (*p* = 0.001)-fed mice (Figure 3b and Figure 4a). Since 3-metylhistidine has been recognized as a biomarker of muscle degradation, this result suggested that replacement by bee larva-derived protein did not cause amino acid deficiency. Indeed, gastrocnemius muscle weight was significantly higher in 30% bee- than in 15% casein-fed mice (*p* = 0.043), but did not differ between 15% casein- and 30% casein-fed mice, also implying that the replacement of bee larva protein from casein did not result amino acid deficiency and cause muscle degradation (Figure 4b). Consistently, results of the wire hang test also implied that muscle degradation was not found in bee larva protein-fed mice (Figure 4c). We further analyzed muscle regeneration-related genes to clarify bee larva’s muscle regeneration ability. Results showed that muscle regeneration genes *Myod* and *Myogenin* did not differ among groups (Figure 4d,e).

### 3.5. Plasma Nicotinamide Was Increased in Bee Larva-Fed Mice

**Figure 5:**
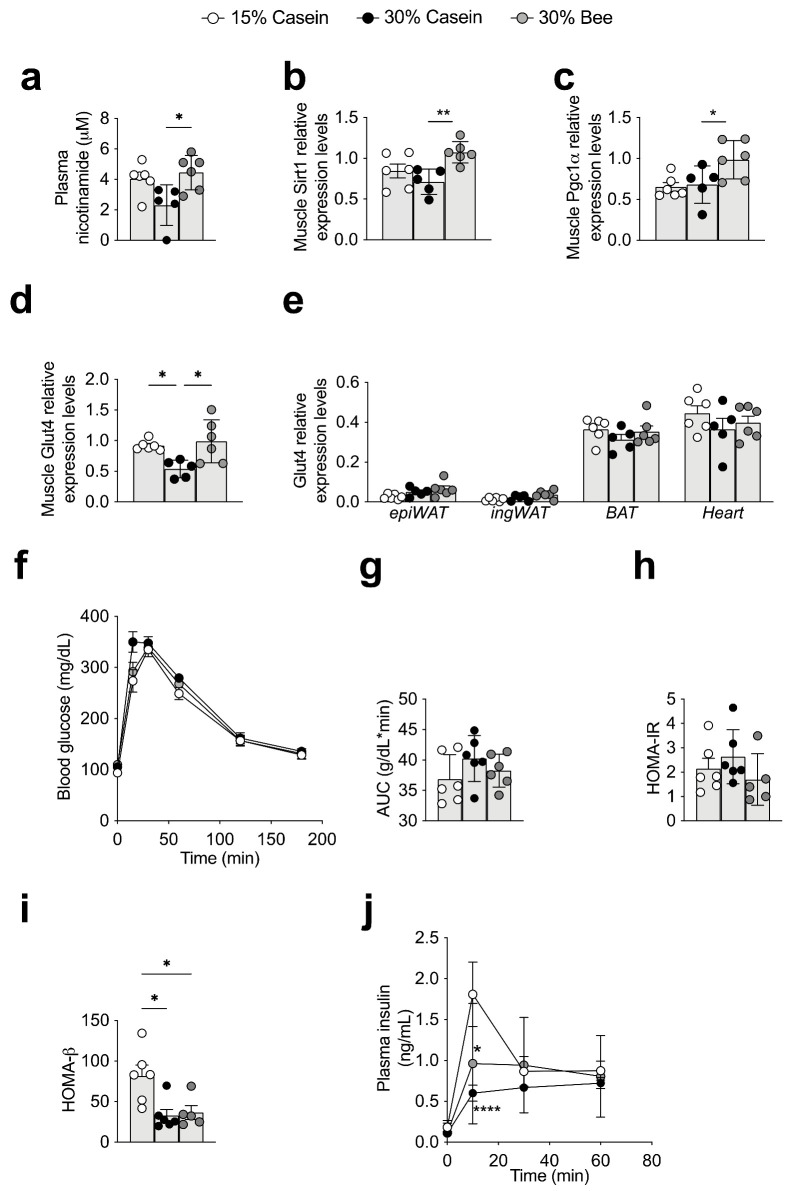
Effects of bee larva protein on glucose metabolism. (**a**–**j**), Analysis of the mice used in Figure 1. (**a**) Plasma nicotinamide levels. (**b**) Relative mRNA expression levels of muscle *Sirt1.* (**c**) Relative mRNA expression levels of muscle *Pgc1α.* (**d**) Relative mRNA expression levels of muscle *Glut4.* (**e**) Relative mRNA expression levels of *Glut4* in epididymal white adipose tissue (epiWAT), inguinal white adipose tissue (ingWAT), brown adipose tissue (BAT), and heart. (**f**) Oral glucose tolerance test (OGTT) after an overnight fast 12 weeks after initiation of treatment. Glucose was administered via gavage at a dose of 1.5 g/kg body weight. (**g**) Area under the curve (AUC) of glucose excursion during the OGTT in panel f. (**h**) HOMA-IR during the OGTT in panel f. (**i**) HOMA-β during the OGTT in panel f. (**j**) Plasma insulin levels during the first hour of the OGTT in panel f. Data are mean ± s.e.m. * *p* ≤ 0.05, ** *p* ≤ 0.01, **** *p* ≤ 0.0001. *p* values were calculated using ANOVA and post hoc tests.

We found that plasma nicotinamide was significantly increased in mice fed 30% bee compared with those fed 30% casein (*p* = 0.021) (Figure 5a), and enrichment analysis using whole plasma metabolite consistently enhanced the nicotinamide pathway (Figure 3c). According to a previous study, nicotinamide improves obesity or type 2 diabetes through the NAD-Sirtuin signaling mechanism [34]; therefore, we further analyzed activities in muscle and adipose tissue, which are important tissues for controlling body weight or blood glucose control.

### 3.6. Bee Larva Increased Glucose Uptake to Muscle

In muscle, the relative gene expression levels of *Sirt1* were significantly higher in 30% bee-fed mice than in 30% casein-fed mice (*p* = 0.009) (Figure 5b). The Sirtuin1 regulating pathway *Pgc1α* was also upregulated in mice fed 30% bee compared with those fed 30% casein (*p* = 0.031) (Figure 5c). In addition, the muscle of the 30% bee diet-fed mice showed higher *Glut4* expression levels than that of 30% casein- (*p* = 0.013) or 15% casein-fed mice (*p* = 0.042), implying that glucose intake to the muscle was increased in 30% bee-fed mice (Figure 5d,e).

To understand how those differences of gene expressions affect actual blood glucose maintenance, we conducted an oral glucose tolerance test (OGTT). The results of OGTT showed that two hours later, blood glucose levels were lower than 200 mg/dL in all groups, which implied that there was no evidence of type 2 diabetes in any of the groups. Regarding protein quantity and quality, area under the curve (AUC) or homeostasis model assessment insulin resistance (HOMA-IR) [35,36,37] were not different among the diet groups (Figure 5f–h). However, HOMA-β calculated from fasting baseline blood glucose and insulin levels showed that insulin secretion was reduced with higher protein levels, regardless of it being casein (*p* = 0.011) or bee larva protein (*p* = 0.024) (Figure 5i). This was consistent with the insulin levels during the OGTT (Figure 5j).

### 3.7. Bee Larva Stimulate Thermogenesis in Brown and White Adipose Tissue

**Figure 6:**
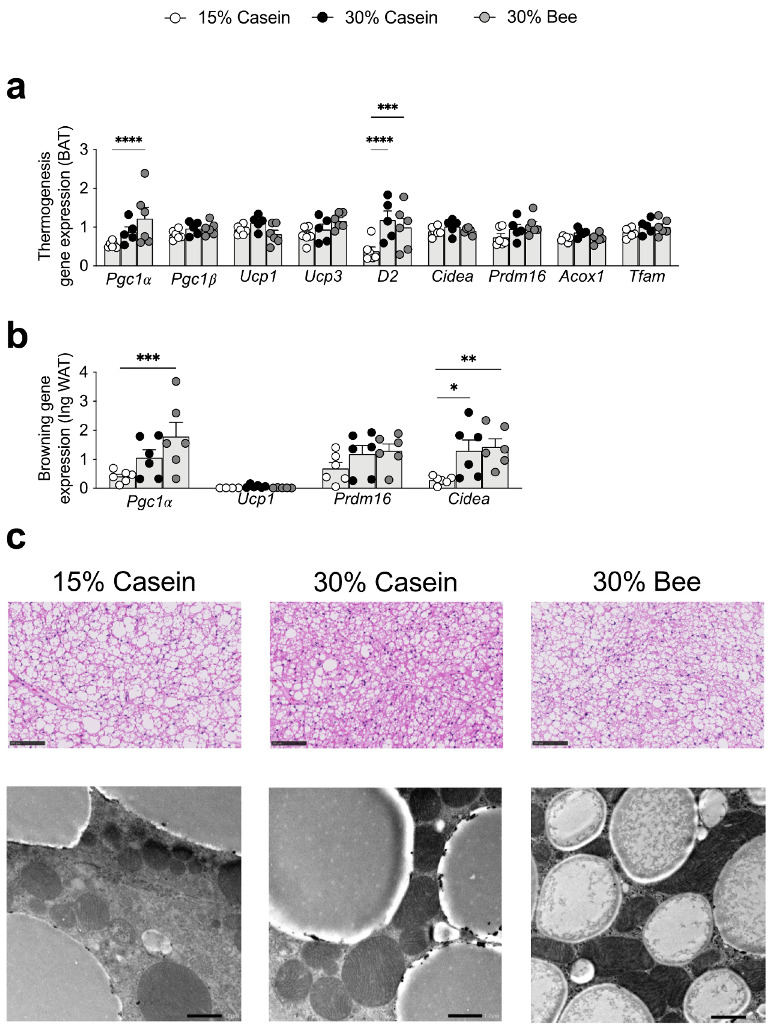
Effects of bee larva protein on lipid metabolism. (**a**–**c**), Analysis of the mice used in Figure 1. (**a**) Relative mRNA expression levels involved in thermogenesis in brown adipose tissue (BAT). (**b**) Relative mRNA expression levels involved in thermogenesis inguinal white adipose tissue (ingWAT). (**c**) Representative images of BAT stained with H&E. Magnification bar, 100 μm. Representative image of transmission electron microscopy analysis of BAT. Scale bar, 1 μm. Data are mean ± s.e.m. * *p* ≤ 0.05, ** *p* ≤ 0.01, *** *p* ≤ 0.001, **** *p* ≤ 0.0001. *p* values were calculated using ANOVA and post hoc tests.

Histological analysis revealed that brown fat showed more miniaturized and polycystic adipocytes in high protein groups (30% casein, 30% bee) than in the 15% casein protein group and was more prominent in 30% bee (Figure 6c).

We observed the mitochondria of brown adipocytes by electron microscopy (Figure 6c), and found that there were smaller fat droplets and increased mitochondria in the 30% bee group.

*Pgc1α*, which are sirt1 regulatory genes, were upregulated in the brown adipose tissue (BAT) of 30% bee-fed mice (*p* < 0.0001) (Figure 6a). This was also observed in inguinal white adipose tissue (*p* = 0.0009 for *Pgc1α* and *p* = 0.006 for *Cidea*) (Figure 6b), suggesting that 30% bee administration activated the browning of white adipose tissue.

## 4. Discussion

There is a growing food crisis, especially a shortage of protein sources, due to environmental change and population growth [38,39,40]; therefore, sustainable solutions are needed to meet the food requirements of the growing population [2]. Edible insect diets have attracted much attention as a sustainable protein source due to their high conversion rate and low energy requirements for production [8,9,10].

On the other hand, previous studies have shown that simply changing the protein source can cause changes in energy metabolism [22,23,24,25,26]. Therefore, it is necessary to investigate the effects of edible insect-derived proteins in vivo in order to achieve a sustainable protein supply. In this study, we replaced casein, a conventional animal protein, with bee larval-derived protein and investigated its effects on amino acid levels and metabolic signals in vivo.

Edible insects are rich in protein. According to previous studies, the protein content on dry matter basis of edible insects ranges from 35.3% in isoptera (termites) to 61.3% in orthoptera (crickets, grasshoppers, locusts) [41]. In a previous study on worker honey bee larvae, *Apis mellifera ligustica*, the protein content on dry matter basis was 35.3% [42] and the bee larva-derived protein we used in this study consists of 48.5% of dry matter.

One of the most important properties of consuming different protein sources is that essential amino acids cannot be produced by the human body and therefore must be obtained from food [43,44]. A previous study reported that edible insects are low in the amino acids methionine and cysteine and high in lysine and threonine [45], whereas other previous research also showed that edible insects generally meet the World Health Organization’s amino acid requirements [41]. A previous study found that leucine and lysine were high in worker honey bee larvae, *Apis mellifera ligustica* [42]. Lysine is a limiting amino acid source and providing a sufficient lysine supply is one of the most critical factors for achieving consideration as a sustainable protein source [46]. Lysine synthesizes carnitine, and carnitine is required to transport fatty acids to the mitochondria for beta-oxidation. According to the previous studies, proteins derived from honey bee larva met all the levels of the presumed essential amino acids in the protein pattern recommended by FAO/WHO/UNU (2007) [42]. In addition to the presence of these essential amino acids, an appropriate balance of essential and non-essential amino acids and other nitrogen-containing compounds is necessary for effective utilization of dietary protein [42]. Among the non-essential amino acids, glutamate/glutamine was high in bee larva-derived protein, which was consistent with previous studies on other edible insects [42].

The quality of edible insect proteins needs to be assessed using in vivo studies [47]. Although essential amino acids must be consumed throughout the diet, replacing all dietary protein with bee larvae protein during the 22-week treatment period did not result in a deficiency of essential amino acids in blood and liver. Our results showed that for the essential amino acids, lysine was high, and for the non-essential amino acids, glutamine was high in the blood of mice fed bee larva protein. Compared with results of previous study, our study showed that lysine and glutamine, which was rich in bee larva protein itself, were present in high concentrations in blood on mice fed bee larva protein.

In addition, we observed that bee larva protein did not stimulate muscle degradation. The muscle degradation caused by amino acid deficiency is one of the most critical risks of using alternative protein sources and risk factor for sarcopenia [48]. Our results showed that muscle weight or muscle strength did not change, and a possible biomarker for muscle degradation, 3-methylhistidine [49,50,51], decreased in bee larva-derived protein-fed mice. No weight loss was also observed during the 22-week treatment period in the mice, suggesting that the bee larvae-derived protein does not cause significant side effects.

High-throughput and comprehensive analysis of intracellular metabolites is suitable for revealing the connection of biochemical networks and enhance a systems-level understanding of the cell [52]. In this study, we performed comprehensive metabolite analysis by CE-TOF/MS, which is suitable for the measurement of amino acids and water-soluble small-molecule metabolites [53]. Results of the comprehensive metabolite analysis showed that nicotinamide was increased in bee larva-derived protein-fed mice. Since nicotinamide is involved in the NAD (nicotinamide adenine dinucleotide) pathway, we investigated associations with the longevity gene *Sirt1*, which codes for an NAD-dependent deacetylase. Sirt1 is involved in muscle hypertrophy, blood glucose uptake, and browning in adipocytes [47]. In muscle, gene expression levels of insulin responsive blood glucose transporter *Glut4* was significantly increased in bee larva protein-fed mice. These results suggested that *Glut4* is a downstream gene of Sirt1 signaling and that the bee larvae-derived protein activates Sirt1 signaling, which in turn increases *Glut4* gene expression in muscle. To further confirm the effect of blood glucose control, OGTT was performed, but the results did not change among the groups. In the present study, we did not use a diet with 60% kcal fat, which is used in diet-induced obesity models, but the fat was set at 30%, which is close to the average value in the US NHANES study [30,31]; therefore, the effect of improving diabetes could not be clearly clarified from the OGTT results. Future studies using obesity or diabetic models are also necessary to clarify effects of bee larvae-derived protein on diabetes.

In adipocytes, gene expression levels of thermogenesis-related genes were significantly increased in bee larva protein-fed mice. Results of histology analysis imply that mitochondria function might improve in bee larva-fed mice.

It is possible that not only the amino acid composition, but the lipid composition of bee larvae also affected the phenotypic differences between muscle and adipose tissues in this study. In addition, this study used starch as an adjuster for the carbohydrate content to replace the protein source as conducted in a previous study [23]. However, bee larva-derived carbohydrate is not present in equal amounts as to the amount of starch in controls for this study. Recent research suggested that the type of carbohydrate is one of the important factors for affecting metabolic effects [54]. Further studies are needed to clarify the detailed mechanisms between edible insect compounds that affect metabolism.

This dataset has showed that edible insect-derived proteins have beneficial effects for health. It also clarified that edible insects, such as bee larva, which can be a potential source of protein, did not cause a shortage of amino acids and muscle degradation in vivo. Replacement of casein by bee larva-derived protein may change glucose uptake in muscle, and upregulate thermogenesis in adipose tissue. As such, bee larvae have the potential to be a sustainable and healthy protein source in the future.

## Supplementary Materials

The following are available online at https://www.mdpi.com/article/10.3390/foods10112642/s1. Table S1: Ingredients of the diets, Table S2: Primer sequences for PCR.

## References

[1] Wu G. (2009). Amino acids: Metabolism, functions, and nutrition. Amino Acids.

[2] Willett W., Rockstrom J., Loken B., Springmann M., Lang T., Vermeulen S., Garnett T., Tilman D., DeClerck F., Wood A. (2019). Food in the Anthropocene: The EAT-Lancet Commission on healthy diets from sustainable food systems. Lancet.

[3] Foley J.A., Defries R., Asner G.P., Barford C., Bonan G., Carpenter S.R., Chapin F.S., Coe M.T., Daily G.C., Gibbs H.K. (2005). Global consequences of land use. Science.

[4] Vermeulen S.J., Campbell B.M., Ingram J.S. (2012). Climate change and food systems. Annu. Rev. Environ. Resour..

[5] Steffen W., Richardson K., Rockstrom J., Cornell S.E., Fetzer I., Bennett E.M., Biggs R., Carpenter S.R., de Vries W., de Wit C.A. (2015). Sustainability. Planetary boundaries: Guiding human development on a changing planet. Science.

[6] Molden D. (2007). Comprehensive Assessment of Water Management in Agriculture. Water for Food, Water for Life: A Comprehensive Assessment of Water Management in Agriculture.

[7] Verneau F., Amato M., La Barbera F. (2021). Edible Insects and Global Food Security. Insects.

[8] Parodi A., Leip A., de Boer I.J.M., Slegers P.M., Ziegler F., Temme E.H.M., Herrero M., Tuomisto H., Valin H., van Middelaar C.E. (2018). The potential of future foods for sustainable and healthy diets. Nat. Sustain..

[9] Huis A.V., Itterbeeck J.V., Klunder H., Mertens E., Halloran A., Muir G., Vantomme P. (2013). Edible insects: Future prospects for food and feed security.

[10] Huis A.V. (2015). Edible insects contributing to food security?. Agric. Food Secur..

[11] OECD (2017). Obesity Update. www.oecd.org/health/obesity-update.htm.

[12] United Nations Population Division (2017). World Population Prospects: The 2019 Revision.

[13] Cesard N., Komatsu S., Iwata A. (2015). Processing insect abundance: Trading and fishing of zazamushi in Central Japan (Nagano Prefecture, Honshu Island). J. Ethnobiol. Ethnomed..

[14] Nonaka K. (2009). Feasting on insects. Entomol. Res..

[15] Mitsuhashi J. (1997). Insects as traditional foods in Japan. Ecol. Food Nutr..

[16] Mitsuhashi J. (2008). The Complete World of Entomophagy.

[17] Nonaka K., Patrick B., Durst D.V.J., Robin N.L., Kenichi S. (2010). Cultural and commercial roles of edible wasps in Japan. Forest Insects as Food: Humans Bite Back.

[18] Kim T.K., Yong H.I., Kim Y.B., Kim H.W., Choi Y.S. (2019). Edible Insects as a Protein Source: A Review of Public Perception, Processing Technology, and Research Trends. Food Sci. Anim. Resour..

[19] Kageyama M., Li K., Sun S., Xing G., Gao R., Lei Z., Zhang Z. (2018). Anti-tumor and anti-metastasis activities of honey bee larvae powder by suppressing the expression of EZH2. Biomed. Pharmacother..

[20] Jensen A.B., Evans J., Jonas-Levi A., Benjamin O., Martinez I., Dahle B., Roos N., Lecocq A., Foley K. (2019). Standard methods for Apis mellifera brood as human food. J. Apic. Res..

[21] Dietemann V., Ellis J.D., Neumann P. (2013). The COLOSS BEEBOOK Volume II, Standard methods for *Apis mellifera* pest and pathogen research: Introduction. J. Apic. Res..

[22] Hashidume T., Kato A., Tanaka T., Miyoshi S., Itoh N., Nakata R., Inoue H., Oikawa A., Nakai Y., Shimizu M. (2016). Single ingestion of soy beta-conglycinin induces increased postprandial circulating FGF21 levels exerting beneficial health effects. Sci. Rep..

[23] Wojcik J.L., Devassy J.G., Wu Y., Zahradka P., Taylor C.G., Aukema H.M. (2016). Protein source in a high-protein diet modulates reductions in insulin resistance and hepatic steatosis in fa/fa Zucker rats. Obesity (Silver Spring).

[24] Mariotti F. (2019). Animal and Plant Protein Sources and Cardiometabolic Health. Adv. Nutr..

[25] Berrazaga I., Micard V., Gueugneau M., Walrand S. (2019). The Role of the Anabolic Properties of Plant- versus Animal-Based Protein Sources in Supporting Muscle Mass Maintenance: A Critical Review. Nutrients.

[26] Levine M.E., Suarez J.A., Brandhorst S., Balasubramanian P., Cheng C.W., Madia F., Fontana L., Mirisola M.G., Guevara-Aguirre J., Wan J. (2014). Low protein intake is associated with a major reduction in IGF-1, cancer, and overall mortality in the 65 and younger but not older population. Cell Metab..

[27] Kim T.K., Yong H.I., Jang H.W., Kim Y.B., Choi Y.S. (2020). Functional Properties of Extracted Protein from Edible Insect Larvae and Their Interaction with Transglutaminase. Foods.

[28] van Huis A. (2020). Nutrition and health of edible insects. Curr. Opin. Clin. Nutr. Metab. Care.

[29] Jeong H., Shin K. (2020). What Is Required for Edible Insects to Become Medical Food? From a Health Professionals and Caregivers’ Perspective. Insects.

[30] Berryman C.E., Lieberman H.R., Fulgoni V.L., Pasiakos S.M. (2018). Protein intake trends and conformity with the Dietary Reference Intakes in the United States: Analysis of the National Health and Nutrition Examination Survey, 2001–2014. Am. J. Clin. Nutr..

[31] Austin G.L., Ogden L.G., Hill J.O. (2011). Trends in carbohydrate, fat, and protein intakes and association with energy intake in normal-weight, overweight, and obese individuals: 1971–2006. Am. J. Clin. Nutr..

[32] Sugimoto M., Wong D.T., Hirayama A., Soga T., Tomita M. (2010). Capillary electrophoresis mass spectrometry-based saliva metabolomics identified oral, breast and pancreatic cancer-specific profiles. Metabolomics.

[33] Xia J., Wishart D.S. (2011). Web-based inference of biological patterns, functions and pathways from metabolomic data using MetaboAnalyst. Nat. Protoc..

[34] Mitchell S.J., Bernier M., Aon M.A., Cortassa S., Kim E.Y., Fang E.F., Palacios H.H., Ali A., Navas-Enamorado I., Di Francesco A. (2018). Nicotinamide Improves Aspects of Healthspan, but Not Lifespan, in Mice. Cell Metab..

[35] Turner R.C., Holman R.R., Matthews D., Hockaday T.D., Peto J. (1979). Insulin deficiency and insulin resistance interaction in diabetes: Estimation of their relative contribution by feedback analysis from basal plasma insulin and glucose concentrations. Metabolism.

[36] Matthews D.R., Hosker J.P., Rudenski A.S., Naylor B.A., Treacher D.F., Turner R.C. (1985). Homeostasis model assessment: Insulin resistance and beta-cell function from fasting plasma glucose and insulin concentrations in man. Diabetologia.

[37] Rudenski A.S., Matthews D.R., Levy J.C., Turner R.C. (1991). Understanding “insulin resistance”: Both glucose resistance and insulin resistance are required to model human diabetes. Metabolism.

[38] World Food Programme, United Nations 2020—Global Report on Food Crises. https://www.wfp.org/publications/2020-global-report-food-crises.

[39] McLaren D.S. (2000). The great protein fiasco revisited. Nutrition.

[40] McLaren D.S. (1974). The great protein fiasco. Lancet.

[41] Rumpold B.A., Schluter O.K. (2013). Nutritional composition and safety aspects of edible insects. Mol. Nutr. Food Res..

[42] Ghosh S., Jung C., Meyer-Rochow V.B. (2016). Nutritional value and chemical composition of larvae, pupae, and adults of worker honey bee, Apis mellifera ligustica as a sustainable food source. J. Asia Pac. Entomol..

[43] Lizarazo C.I., Lampi A.M., Liu J.W., Sontag-Strohm T., Piironen V., Stoddard F.L. (2015). Nutritive quality and protein production from grain legumes in a boreal climate. J. Sci. Food Agric..

[44] Foyer C.H., Lam H.M., Nguyen H.T., Siddique K.H.M., Varshney R.K., Colmer T.D., Cowling W., Bramley H., Mori T.A., Hodgson J.M. (2016). Neglecting legumes has compromised human health and sustainable food production. Nat. Plants.

[45] DeFoliart G.R. (1992). Insects as human food: Gene DeFoliart discusses some nutritional and economic aspects. Crop Prot..

[46] Leinonen I., Iannetta P.P.M., Rees R.M., Russell W., Watson C., Barnes A.P. (2019). Lysine Supply Is a Critical Factor in Achieving Sustainable Global Protein Economy. Front. Sustain. Food Syst..

[47] Liang F., Kume S., Koya D. (2009). SIRT1 and insulin resistance. Nat. Rev. Endocrinol..

[48] Fujita S., Volpi E. (2006). Amino acids and muscle loss with aging. J. Nutr..

[49] McKeran R.O., Halliday D., Purkiss P., Royston P. (1979). 3-Methylhistidine excretion as an index of myofibrillar protein catabolism in neuromuscular disease. J. Neurol. Neurosurg. Psychiatry.

[50] Trappe T., Williams R., Carrithers J., Raue U., Esmarck B., Kjaer M., Hickner R. (2004). Influence of age and resistance exercise on human skeletal muscle proteolysis: A microdialysis approach. J. Physiol..

[51] Mussini E., Cornelio F., Dworzak F., Cotellessa L., Morandi L., Colombo L., De Ponte G., Marcucci F. (1983). Content of methylhistidines in normal and pathological human skeletal muscles. Muscle Nerve.

[52] Soga T., Ohashi Y., Ueno Y., Naraoka H., Tomita M., Nishioka T. (2003). Quantitative metabolome analysis using capillary electrophoresis mass spectrometry. J. Proteome Res..

[53] Hirayama A., Ikeda S., Sato A., Soga T. (2019). Amino Acid Analysis by Capillary Electrophoresis-Mass Spectrometry. Methods Mol. Biol..

[54] Wali J.A., Milner A.J., Luk A.W.S., Pulpitel T.J., Dodgson T., Facey H.J.W., Wahl D., Kebede M.A., Senior A.M., Sullivan M.A. (2021). Impact of dietary carbohydrate type and protein-carbohydrate interaction on metabolic health. Nat. Metab..

