# Supplementary material for: Metabolic Effects of Bee Larva-Derived Protein in Mice: Assessment of an Alternative Protein Source
Source: Foods. 2021 Nov 1;10(11):2642. doi: 10.3390/foods10112642 (PMC8624207; doi:10.3390/foods10112642)
Supplement: Supplementary file 1 [file foods-10-02642-s001.zip › foods-1393286-supplementary.pdf]

Supplemental Table S1. Ingredients of the diets.

	15% Casein	30% Casein	30% Bee
Ingredients			
Casein	195	390	0
Bee larva	0	0	710
β-cornstarch	375	180	50
α-cornstarch	132	132	132
Sucrose	100	100	100
Lard	150	150	0
Cellulose	50	50	50
AIN-93M mineral mix	35	35	35
AIN-93VX vitamin mix	10	10	10

Supplemental Table S2. Primer sequences for PCR.

Gene	Forward	Reverse
β-actin	CATCCGTAAAGACCTCTATGCCAAC	ATGGAGCCACCGATCCACA
Pgc1α	AAGGGCCAAACAGAGAGAGA	GCGTTGTGTCAGGTCTGATT
Pgc1β	AGATGAAGATCCAAGCTGCCCACA	TCCTCCTCCATTGGCTTGTATGGA
Ucp-1	GGCCCTTGTAAACAACAAAATAC	GGCAACAAGAGCTGACAGTAAAT
Ucp-3	CTCTGCACTGTATGCTGAAGATG	CACGTTCCAAGCTCCCAGA
D2	TTCTGAGCCGCTCCAAGT	GGAGCATCTTCACCCAGTTT
Cidea	TGCTCTTCTGTATCGCCCAGT	GCCGTGTTAAGGAATCTGCTG
Prdm16	CAGCACGGTGAAGCCATTC	GCGTGCATCCGCTTGTG
Acox-1	TTCTACCAATCTGGCTGCAC	GTGGGTGGTATGGTGTCGTA
Tfam	ATGTCTCCGGATCGTTTCAC	CCAAAAAGACCTCGTTCAGC
Sirt1	GCACCGAGGAACTACCTGAT	CAGCATCTTGCCTGATTTGT
Glut4	ATCAACGCCCCACAGAAA	GAGAGCCCAAAGGGTAGTGA
MyoD	GTCGTAGCCATTCTGCCG	AGCACTACAGTGGCGACTCA
Myogenin	GTGGGAGTTGCATTCACTGG	CTACAGGCCTTGCTCAGCTC
